# Synergistic promotion between modified carbon cloth electrode and supramolecular gel polymer electrolyte enables flexible energy storage

**DOI:** 10.1039/d6ra02954j

**Published:** 2026-07-03

**Authors:** Pengzhen Wang, Wenzhe Xue, Pengyu Han, Xiuming Wu, Xinwang Song, Chunxiao Zhang, Zhaoting Meng

**Affiliations:** a Shandong Key Laboratory of Green Electricity & Hydrogen Science and Technology, School of Chemical Engineering, Shandong Institute of Petroleum and Chemical Technology Dongying 257061 China mztuser@163.com

## Abstract

The development of high-performance flexible supercapacitors necessitates synergistic design between electrodes and electrolytes. Herein, a flexible supramolecular gel supercapacitor (FSGSC) was constructed by integrating a polypyrrole modified carbon cloth (PPy/CC) electrode with a sodium alginate/acrylamide acrylic copolymer supramolecular gel polymer electrolyte. The PPy/CC electrode offers a high areal capacitance of 3600.0 mF cm^−2^, while the interpenetrating network gel ensures robust mechanical properties and tunable ionic conductivity. A crucial synergistic promotion is unveiled, where the device performance strongly depends on the NaCl concentration within the gel electrolyte. An optimal salt content yields an exceptional specific capacitance of 991.1 mF cm^−2^, low resistance, and efficient kinetics. This work highlights the importance of harmonized component engineering for advanced flexible energy storage.

## Abstract 2

The development of high-performance flexible supercapacitors necessitates synergistic design between electrodes and electrolytes.

## Graphical abstract

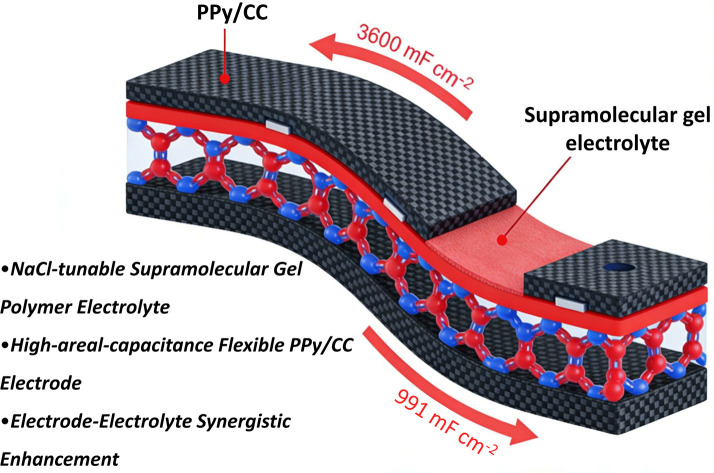

## 1. Introduction

The burgeoning field of wearable and portable electronics has triggered an unprecedented demand for flexible, lightweight, and high-performance energy storage devices.^1–5^ Among various candidates, supercapacitors have garnered significant attention due to their high power density, rapid charge–discharge capabilities, and long cycle life.^6–9^ However, conventional supercapacitors, often rigid and bulky, fall short in meeting the mechanical compliance required for flexible applications.^10–15^ Therefore, the development of all-solid-state flexible supercapacitors, which integrate flexible electrodes and solid-state electrolytes, has become a pivotal research frontier.^16–21^ The ultimate performance of these devices is not solely determined by the electrode or electrolyte alone but hinges critically on the synergistic interplay between them.^22–25^ A key challenge lies in harmonizing high electrochemical activity and mechanical flexibility of the electrode with superior ionic conductivity and robust interfacial contact offered by the electrolyte, thereby creating an integrated system with enhanced energy storage capabilities and mechanical durability.

The electrode and electrolyte serve as the core components dictating the performance of flexible supercapacitors. On the electrode side, carbon cloth (CC) is a widely used flexible substrate owing to its excellent conductivity, woven structure, and chemical stability.^26–31^ Nevertheless, the pristine CC typically exhibits limited charge storage capacity due to its low specific surface area and the absence of faradaic reactions. To address this, surface modification with conductive polymers, such as polypyrrole (PPy), has proven effective. PPy can significantly enhance the capacitance through pseudocapacitive contributions while maintaining flexibility.^32–36^ However, achieving a uniform and adherent PPy coating that fully exploits the conductive network of CC without compromising interfacial stability remains a task. On the electrolyte side, liquid electrolytes pose risks of leakage and corrosion, making gel polymer electrolytes (GPEs) the preferred choice for flexible devices.^37–40^ Supramolecular GPEs, particularly those based on interpenetrating polymer networks (IPNs) like sodium alginate (SA) and poly(acrylamide-*co*-acrylic acid) P(AM-AA), offer intriguing prospects.^41–45^ They combine the mechanical integrity of a cross-linked network with high ionic conductivity and excellent water retention. Despite individual advancements, the synergistic effects between a meticulously engineered PPy/CC electrode and a tunable supramolecular gel electrolyte have not been thoroughly explored, especially regarding how the ionic environment of the gel influences the charge storage dynamics at the electrode–electrolyte interface.

Herein, this work presents a holistic strategy for constructing a high-performance flexible supramolecular gel supercapacitor (FSGSC) *via* the synergistic integration of a modified carbon cloth electrode and a tailored supramolecular gel electrolyte. A flexible PPy/CC composite electrode was fabricated through *in situ* polymerization of pyrrole, resulting in a dense, uniform conductive coating that enhances electroactivity and provides an exceptional areal capacitance. Concurrently, a supramolecular gel electrolyte was synthesized from SA and P(AM-AA), with its ionic conductivity precisely tuned by varying the NaCl content. The assembled symmetric FSGSC leverages the synergistic promotion between the two components: the pseudocapacitive PPy/CC electrode offers abundant active sites for efficient charge storage, while the ion-conductive gel electrolyte ensures effective ion transport and intimate interfacial contact, all within a fully flexible sandwich architecture. This work systematically investigated the correlation between the NaCl concentration in the gel electrolyte and the overall electrochemical performance of the FSGSC. It was demonstrated that an optimal salt content (0.2 g in our preparation) is critical for maximizing the specific capacitance, achieving a remarkable value of 991.1 mF cm^−2^, while maintaining low resistance and excellent charge transfer kinetics. This work underscores the importance of coordinated electrode and electrolyte engineering and provides a promising pathway for developing advanced flexible energy storage systems.

## 2. Experimental section

### 2.1 Preparation of flexible electrodes

Carbon cloth (CC) was cut into 1 cm × 1 cm pieces and subjected to ultrasonic cleaning in ethanol and deionized water for 15 minutes each to remove surface oils and particulate contaminants. The samples were then dried at 60 °C for 2 hours in an electric oven. After drying, the carbon cloth was immersed in concentrated nitric acid for 1 hour to introduce oxygen-containing functional groups on its surface. A mixed solvent consisting of 25 mL of deionized water and 25 mL of ethanol was prepared. Then, 4.0 g of FeCl_3_ was dissolved in the solvent under magnetic stirring until complete dissolution. Subsequently, 3.4 g of pyrrole was slowly added dropwise into the solution, and stirring was continued for 10 minutes. The pretreated carbon cloth was immersed in the reaction solution to ensure full infiltration and allowed to stand at room temperature for 24 hours. After polymerization, the CC was removed, thoroughly rinsed with deionized water to terminate the reaction, and dried at 60 °C for 6 hours. The final product was a flexible PPy/CC composite electrode. A 1 mol L^−1^ NaCl aqueous solution was used as the electrolyte.

### 2.2 Preparation of supramolecular gel electrolyte

First, a 3 wt% sodium alginate (SA) solution was obtained by dissolving 0.3 g of SA in distilled water and bringing the total volume to 10 mL under stirring. Then, 1.0 g of acrylamide (AM), 2.0 g of acrylic acid (AA), and 2.0 mL of distilled water were mixed with 4.5 g of the as-prepared SA solution. Different masses of NaCl (0.1 g, 0.2 g, or 0.3 g) and a certain amount of ammonium persulfate (APS) were added as the cross-linking agent and initiator, respectively. After stirring for 1 h, the homogeneous solution was transferred into a sample vial and placed in an oven at 55 °C for 8 h to form the supramolecular gel electrolyte (S-gel_*X*_, where *X* = 0.1, 0.2, or 0.3 g NaCl). For each NaCl concentration, three parallel samples were prepared.

### 2.3 Preparation of flexible supramolecular gel supercapacitor (FSGSC)

A flexible symmetric supercapacitor was assembled in a sandwich configuration using the as-synthesized supramolecular gel electrolyte as the electrolyte and two identical PPy/CC flexible composite electrodes as the electrodes.

### 2.4 Evaluation of energy storage capability

The specific capacitance (*C*), energy density (*E*) and power density (*P*) of FSGSC were calculated by the following equations:1
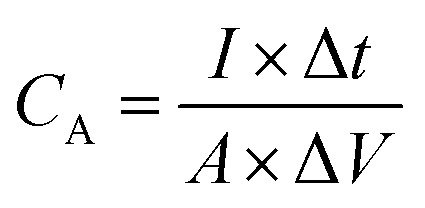
2
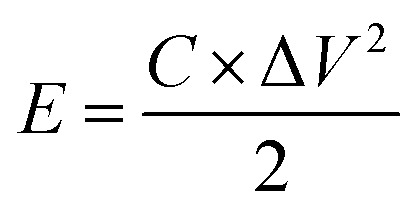
3
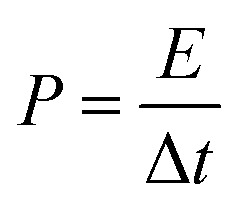
where *A*, *I*, Δ*V*, Δ*t*, and *C* are the quality of active substances, discharge current, potential window, discharge time and specific capacitances of the FSGSC, respectively.

### 2.5 The formula for calculating conductivity is as follows



4

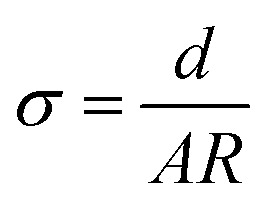

where *σ*, *d*, *A* and *R* represent the conductivity, thickness, effective contact area, and resistance of the ionic gel electrolyte, respectively.

### 2.6 Characterization

The morphology and structure of ionic gel electrolyte were characterized by SEM, FT-IR (Nicolet IS50 instrument, Thermo Fisher), and Raman (LabRAM Odyssey). Electrochemical testing was performed using the CHI-760 E electrochemical workstation (ChenHua, China). The frequency range for electrochemical impedance spectroscopy (EIS) was from 10^−2^ Hz to 10^5^ Hz.

## 3 Results and discussion

**Fig. 1:**
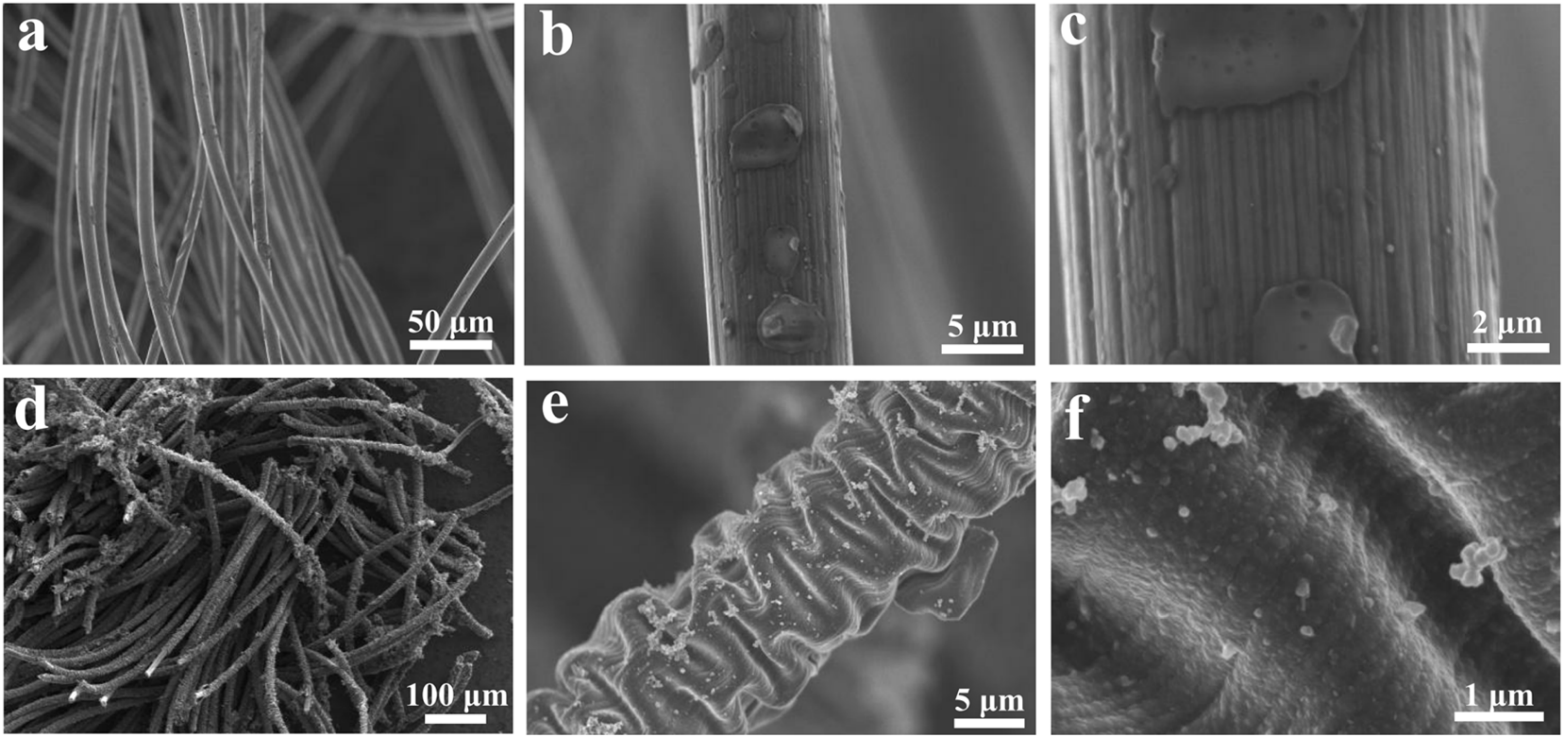
(a–f) SEM images of CC (a–c) and PPy/CC (d and e).

The two carbon cloth samples were further examined by scanning electron microscopy (SEM). Comparative analysis between the polypyrrole (PPy)-modified carbon cloth and the untreated sample revealed distinct morphological differences (Fig. 1). At low magnification, the PPy-treated sample exhibited uniformly coated fibers. Under higher magnification, a dense layer of nanoscale particles or fibrous structures became apparent, confirming the successful deposition of PPy and the formation of a finely structured conductive polymer coating. In contrast, the untreated carbon cloth showed exposed and smooth fiber surfaces at comparable magnifications, with no observable coating. These results demonstrate that PPy modification significantly alters the microstructure of carbon cloth, enhancing its surface complexity and potential for functionalization.

**Fig. 2:**
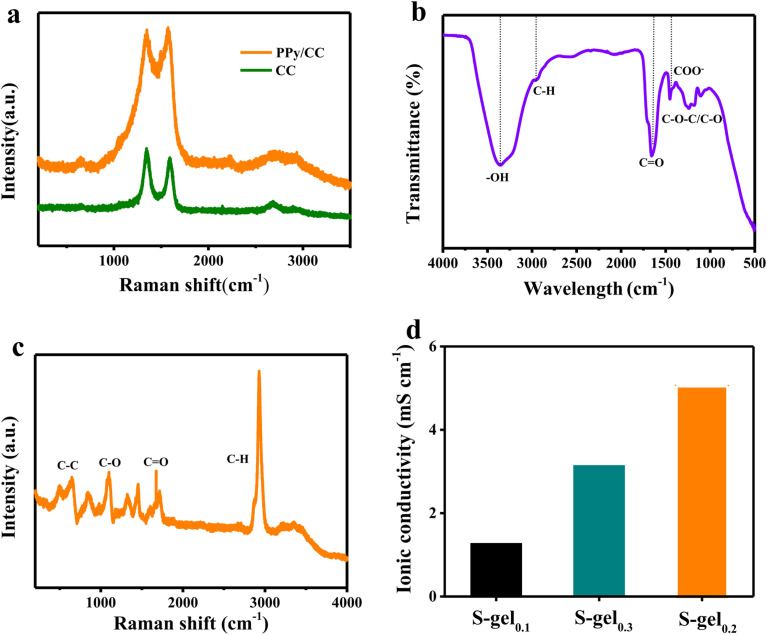
(a–d) Raman spectra of PPy/C and CC (a) FTIR spectra of S-gel_0.2_ (b) Raman spectra of S-gel_0.2_ (c) ionic conductivity of S-gel_*X*_ (*x* = 0.1, 0.2 and 0.3) (d).

The chemical structure of the PPy/CC flexible composite electrode was characterized by Raman spectroscopy. As shown in Fig. 2a, the Raman spectra of CC with and without PPy deposition reveal significant alterations in the structure and surface properties of CC after PPy incorporation. The intensity ratio of the D band (∼1350 cm^−1^), associated with structural defects, to the G band (∼1580 cm^−1^), corresponding to sp^2^-hybridized carbon ordering (*I*_D_/*I*_G_), provides insight into the interfacial mechanism. An increase in *I*_D_/*I*_G_ suggests that PPy introduces additional defects or disorder *via* π–π interactions or chemical bonding, whereas a decrease may result from signal shielding by a uniform PPy overlayer. Furthermore, the appearance of new peaks-attributed to the pyrrole ring stretching vibration at ∼1050 cm^−1^, C=C stretching vibrations between 1400–1500 cm^−1^, and C–N stretching near 1250 cm^−1^-confirms the successful loading of PPy and suggests possible nitrogen doping or interfacial bonding. The C–H stretching vibrations in the 2900–3100 cm^−1^ region further corroborate the presence of PPy, excluding interference from the carbon cloth substrate. A notable decrease in D/G band intensity along with distinct PPy signals indicates uniform and dense coverage of PPy on CC. In contrast, enhanced and broadened D/G bands may reflect interfacial charge transfer or strain-induced distortion. These structural modifications imply that the PPy/CC composite facilitates electron transport through conjugated synergy, while introduced defects or functional groups (*e.g.*, C–N) could enhance electrochemical activity. Moreover, the dense PPy coating may improve the stability of CC in electrolytes, offering a viable strategy for optimizing the performance of supercapacitors, and related devices.

Sodium alginate was uniformly dispersed in deionized water to form a homogeneous solution. Subsequently, acrylamide and acrylic acid monomers were sequentially introduced into this solution and thoroughly dissolved *via* magnetic stirring, ultimately yielding a transparent and stable precursor solution. Following this, a gradient concentration of sodium chloride and ammonium persulfate as the initiator were introduced into the system. After thorough mixing, the solution was transferred into a specific mold. The mold was placed in a constant temperature oven at 55 °C for 8 hours to undergo thermally initiated free radical polymerization, resulting in the formation of a supramolecular gel electrolyte with a dual-network structure. In this system, acrylamide and acrylic acid copolymerize to form poly(acrylamide-acrylic acid) synthetic chains. These chains interpenetrate with the natural sodium alginate polymer chains, creating a stable interpenetrating network structure that endows the hydrogel with excellent mechanical and functional properties. The chemical structure of the supramolecular gel electrolyte was characterized using Fourier Transform Infrared (FTIR) spectroscopy and Raman spectroscopy.

The FTIR spectrum of the supramolecular gel electrolyte is shown in Fig. 2b. The infrared spectrum, obtained in transmission mode, exhibits characteristic absorption features of multiple functional groups: the broad and strong absorption peak around 3300 cm^−1^ is attributed to the stretching vibrations of hydroxyl groups (O–H) and/or amine groups (N–H), indicating the presence of abundant water molecules and/or amine-containing polymers, consistent with its high water content. The weak absorption peak near 2900 cm^−1^ likely stems from C–H stretching vibrations in hydrophobic chain segments. The absorption peaks in the range of 1700–1600 cm^−1^ correspond to carbonyl (C=O) stretching vibrations, suggesting the presence of carboxylic acid groups (–COOH) and/or ester linkages. The doublet near 1600 cm^−1^ and 1400 cm^−1^ can be assigned to the asymmetric and symmetric vibrations of carboxylate ions (COO^−^), respectively, indicating the presence of ionized carboxylic acid structures from alginate. The absorption peak around 1100 cm^−1^ is related to ether bond (C–O–C) or C–O vibrations from hydroxyl groups, potentially originating from the crosslinker. The overall transmittance decreases from high wavenumbers (4000 cm^−1^) towards low wavenumbers (1000 cm^−1^), reflecting the synergistic effects of various functional groups within the hydrogel. In summary, the chemical composition of the supramolecular gel electrolyte is centered around hydrophilic groups, supported by a polymer backbone containing C–O/C–O–C bonds, forming a three-dimensional crosslinked network *via* ester and/or ether linkages, thereby imparting its characteristic high water retention capacity and structural stability. The Raman spectrum of the supramolecular gel electrolyte is shown in Fig. 2c. The Raman spectrum displays characteristic vibrational features of the polymer backbone and functional groups within the range of 500–4500 cm^−1^. In the region of 500–1500 cm^−1^, the C–C skeletal vibration peaks around 800–1200 cm^−1^ indicate the presence of a polyacrylic acid-like or similar polymer backbone structure. The C–O vibration signals between 1000–1300 cm^−1^ may originate from ether bonds in the crosslinker or from hydroxyl/carboxylic acid groups, reflecting the hydrogel's hydrophilicity and crosslinked nature. In the range of 1500–2000 cm^−1^, the peaks around 1600–1800 cm^−1^ correspond to the stretching vibrations of carbonyl groups (C=O). Overall, the spectral features indicate that the molecular structure of the supramolecular gel electrolyte is based on a carbon chain backbone, combined with oxygen-containing functional groups and potential crosslinking sites, which collectively support its high water absorption, flexibility, and functional characteristics. To elucidate the effect of NaCl content on ion transport behavior, the ionic conductivity of different supramolecular gel electrolytes (S-gel_*x*_) was measured, as shown in Fig. 2d. S-gel_0_._1_ (containing 0.1 g NaCl) exhibited the lowest conductivity of approximately 1.28 mS cm^−1^, attributable to the limited number of mobile charge carriers and poor connectivity of the gel network. Increasing the NaCl content to 0.2 g significantly enhanced the conductivity to about 5.05 mS cm^−1^, which is ascribed to the synergistic effect of highly dissociated Na^+^/Cl^−^ ions and the formation of a continuous ion transport network at moderate ionic strength. However, further increasing the NaCl content to 0.3 g led to a decrease in conductivity to approximately 3.15 mS cm^−1^. Excessive salt induces ion association and network densification, which reduce the effective charge carrier density and hinder ion migration pathways, respectively. These results demonstrate that the NaCl content must be carefully optimized to balance charge carrier concentration and gel microstructure; S-gel_0_._2_ achieves the optimal ionic conductivity for this system.

**Fig. 3:**
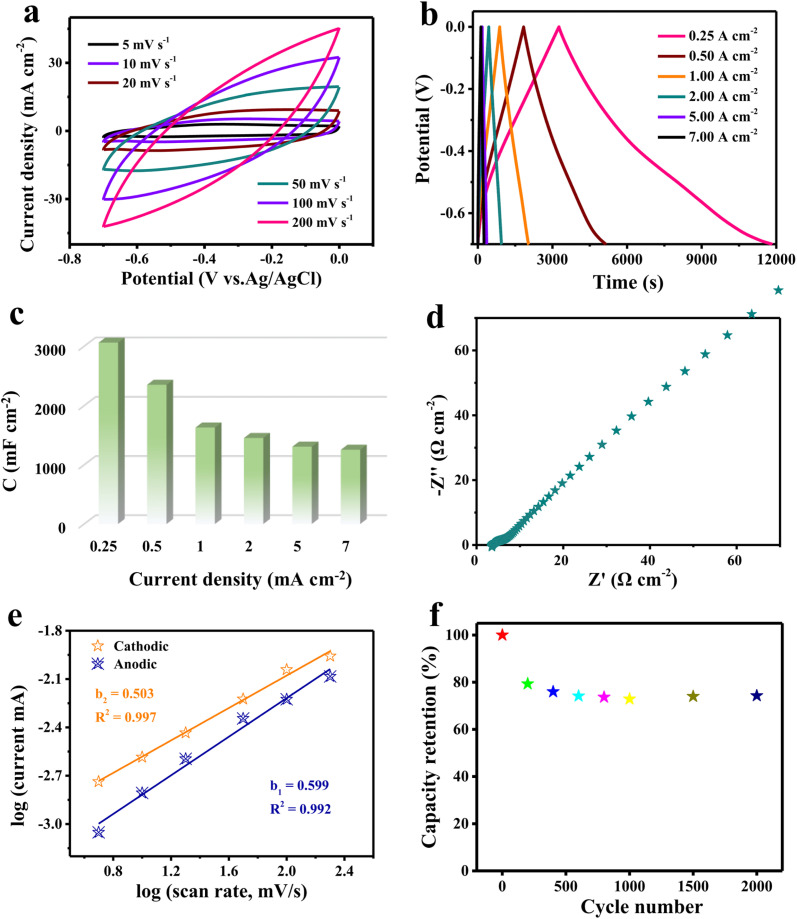
(a–f) CV curves (a) GCD curves (b) capacities (c) Nyquist plots (d) log *i vs.* log *v* plots of PPy/CC electrode at specific peak currents (e) and capacitance retention (f) of PPy/CC.

The electrochemical performance of the PPy/CC composite electrode was evaluated using a three-electrode system with NaCl aqueous solution as the electrolyte, a platinum counter electrode, and a saturated calomel reference electrode. Cyclic voltammetry (CV), galvanostatic charge–discharge (GCD), and electrochemical impedance spectroscopy (EIS) measurements were conducted, and the results are presented in Fig. 3. The CV curves exhibited a stable pseudocapacitive response within a broad potential window of −0.8 to 0.0 V (*vs.* Ag/AgCl), attributable to the synergistic effect between the redox activity of PPy and the conductive carbon cloth substrate. Even at a high scan rate of 200 mV s^−1^, the current response showed no significant decay, indicating rapid charge transfer kinetics. From the GCD curves, the composite exhibits a high areal capacitance of 3600 mF cm^−2^ at a current density of 0.25 A cm^−2^, demonstrating its outstanding charge storage capability. Moreover, the electrode retained superior performance compared to plain carbon cloth even at higher current densities. EIS results revealed lower impedance values across both high- and low-frequency regions for the PPy/CC electrode than for pristine carbon cloth, corroborating its enhanced electrochemical performance. To further investigate the electrochemical kinetics of the PPy/CC electrode, the *b*-values were derived from the logarithmic relationship between peak current and scan rate using CV curves at different scan rates. As shown in Fig. 3e, both *b*-values are approximately 0.5, indicating that the charge/discharge process is dominated by diffusion-controlled kinetics. The PPy/CC electrode was evaluated by cyclic voltammetry at a scan rate of 200 mV s^−1^ for 2000 cycles. After 2000 cycles, the electrode retained 74.27% of its initial capacitance, demonstrating excellent long-term stability (Fig. 3f).

**Fig. 4:**
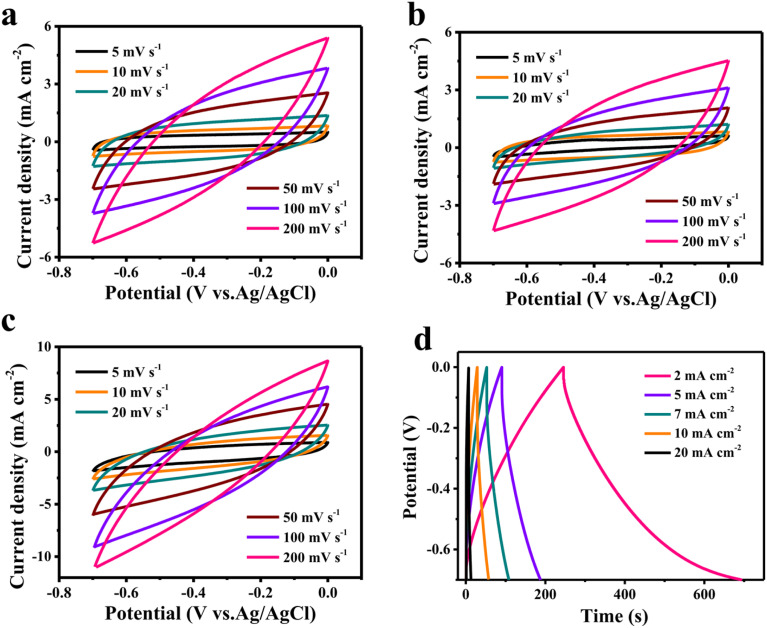
(a and b) CV of FSGSC_0.1_(a) FSGSC_0.3_(b), and FSGSC_0.2_ (c) (c and d) CV and GCD curves of FSGSC_0.2_.

A flexible supercapacitor based on PPy/CC electrode and supramolecular gel electrolyte was assembled. In Fig. 4a–c represent FSGSC_0.1_, FSGSC_0.3_ and FSGSC_0.2_ respectively. As the amount of NaCl added increased from 0.1 g to 0.3 g, the electrochemical performance of the flexible supramolecular gel supercapacitor exhibited an initial increase followed by a decrease. The absolute current density increased from −0.003 A cm^−2^ (0.1 g) to −0.015 A cm^−2^ (0.3 g), indicating that the addition of NaCl reduced the internal resistance by increasing the ionic concentration of the electrolyte, thereby enhancing charge transfer efficiency. The scan rate (5–200 mV s^−1^) positively influenced the current density across all NaCl concentrations, with a more pronounced increase observed at higher concentrations (0.2 g). This suggests that higher salt concentrations may promote surface-controlled charge storage mechanisms, such as electric double-layer capacitance or pseudocapacitance, whereas at lower concentrations (0.1 g), electric double-layer capacitance dominates.

All systems remained stable within the potential window of −0.7 V to 0.0 V (*vs.* Ag/AgCl), with no side reactions induced by varying NaCl concentrations, confirming the structural stability of the material. In summary, a higher NaCl concentration (0.2 g) significantly enhances the energy storage potential of the supramolecular gel electrolyte-based supercapacitor by optimizing ionic conductivity, exposing more active sites, and potentially contributing to pseudocapacitance, making it suitable for the design of high-power electrochemical devices. Fig. 4d illustrates the GCD curve of the device at different current densities, exhibiting a symmetrical triangular shape, which signifies that the device facilitates fast electron transfer and demonstrates good capacitive behavior.

**Fig. 5:**
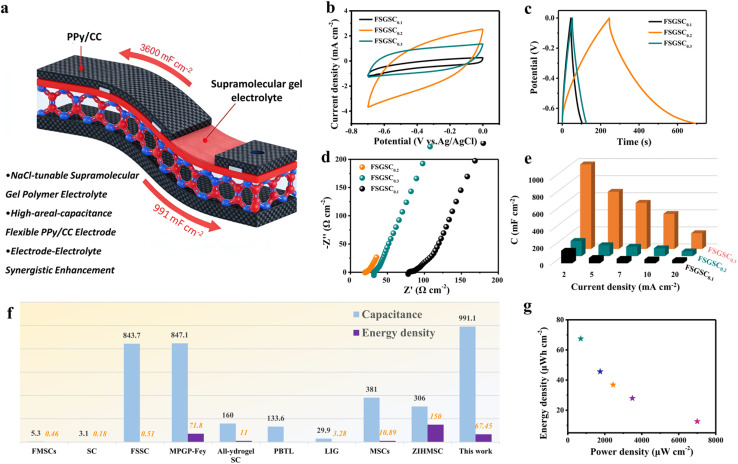
(a)Schematic diagram of flexible supercapacitor based on PPy/CC electrode and supramolecular gel electrolyte. (b–e) Electrochemical performance of FSGSC_*x*_ (*x* = 0.1, 0.2 and 0.3). CV curves at 10 mV s^−1^ (b) GCD curves at 2 mA cm^−2^ (c) Nyquist plots (d) and specific capacitance (e). (f) Electrochemical performances of the specific capacitance and energy density *versus* the power density.^46–54^ (g) Ragone plot of FSGSC_0.10_.

To evaluate and compare the electrochemical performance of the FSGSC devices, their properties were assessed at room temperature, as illustrated in Fig. 5a. Fig. 5b presents a comparison of the cyclic voltammetry (CV) curves for FSGSCs with different NaCl contents, measured at a scan rate of 20 mV s^−1^ within a potential window of 0.7 V. Notably, the FSGSC_0.2_ device exhibited a superior electrochemical response compared to other NaCl variants, which is consistent with the galvanostatic charge–discharge (GCD) behavior shown in Fig. 5c. Fig. 5d displays the electrochemical impedance spectroscopy (EIS) plots of the three devices. The impedance modulus in the high-frequency region was the highest for FSGSC_0.1_, indicating poor electrolyte conductivity and inefficient interfacial charge transfer due to low ionic strength, which correlates directly with its lowest specific capacitance. In contrast, FSGSC_0.2_ showed a lower impedance modulus than both FSGSC_0.1_ and FSGSC_0.3_, suggesting intermediate ionic conductivity and favorable interfacial kinetics at this medium salt concentration. The significantly reduced impedance observed at the highest NaCl content (FSGSC_0.3_) demonstrates enhanced ionic conductivity and lower charge transfer resistance at the electrode/electrolyte interface, aligning with its improved capacitive performance. These EIS results further validate the overall electrochemical advantage of FSGSC_0.2_, characterized by low charge transfer resistance and efficient ion diffusion, forming a coherent evidence chain supporting its highest specific capacitance. As shown in Fig. 5e, the specific capacitances of the devices were calculated from their discharge curves at different current densities. At a current density of 2 mA cm^−2^, the specific capacitance values for FSGSC_0.1_, FSGSC_0.2_, and FSGSC_0.3_ were 148.1, 991.1, and 178.6 mF cm^−2^, respectively. As shown in Fig. 5f and g, the FSGSC device ranks among the most advanced flexible supercapacitors reported to date. Specifically, FSGSC_0.10_ achieves a remarkably high energy density of 67.45 µWh cm^−2^ at a power density of 699.96 µW cm^−2^, outperforming previously reported systems.^46–54^ Owing to its superior flexibility and high energy density, the FSGSC shows great potential as an efficient energy supply system for flexible wearable electronic devices.

## 4 Conclusions

In summary, a high-performance flexible supercapacitor was successfully fabricated by integrating a polypyrrole-modified carbon cloth (PPy/CC) electrode with a supramolecular gel polymer electrolyte. The PPy/CC composite electrode was demonstrated to possess a dense and uniform conductive polymer coating, which introduced structural defects and functional groups, thereby significantly enhancing its electrochemical activity, conductivity, and areal capacitance (reaching 3600 mF cm^−2^). The supramolecular gel electrolyte, featuring an interpenetrating polymer network, provided excellent ionic conductivity and structural stability. A synergistic promotion effect between the modified electrode and the gel electrolyte was confirmed. The electrochemical performance of the assembled flexible supercapacitor was highly dependent on the ionic concentration within the gel electrolyte. An optimal NaCl content of 0.2 g yielded the best performance, achieving a high specific capacitance of 991.1 mF cm^−2^, low internal resistance, and efficient charge transfer kinetics. This work underscores the critical importance of harmonizing electrode modification with electrolyte engineering and presents a promising strategy for developing advanced flexible energy storage devices.

## Conflicts of interest

The authors declare no conflict of interest.

## Data Availability

The authors confirm that the data supporting the findings of this study are available within the article.
